# Development of a chemically defined CHO medium by engineering based on a feed solution

**DOI:** 10.1186/1753-6561-5-S8-P41

**Published:** 2011-11-22

**Authors:** Karlheinz Landauer, Henry Woischnig, Natalie Hepp, Benedikt Greulich, Andreas Herrmann

**Affiliations:** 1 Celonic AG, Basel, Switzerland

## 

The use of chemically defined culture media for production of biopharmaceuticals has become state of the art. In combination with a suitable feed solution chemically defined media are the most potent driving factor for yield improvement in fed-batch processes. Although various chemically defined media are available of-the-shelf and deliver good results, those media are developed with the aim to sustain growth for various host cell lines, and need to be optimized for specific projects.

We describe here the development of CeloCHO, a chemically defined medium for culture of recombinant Chinese hamster ovary cells. The medium was developed by engineering based on an optimised proprietary feed medium, which was developed previously. The approach was straight-forward and linked superior results with minimum investments in medium development.

In a first step of development, the components of the proprietary feed medium were categorized into five component groups: bulk salts, trace elements, vitamins, amino acids, and other components. Necessary components not present in the feed medium were considered while establishing a basic medium formulation based on theoretic considerations of cellular needs in culture (buffer system, adaptation of amino acid concentrations, glucose, growth factors, et cetera). This basic formulation was combined with various x-fold concentrates of the component group stock solutions.

Seven media formulations were developed this way and were used for adaptation in the second step of development. At this stage cell densities up to 4×10^6^ cells per ml in batch experiments were achieved using a CHO-K1 derived recombinant cell line producing a humanized antibody (IgG A). Albeit maximum viable cell density and integral of viable cell density were significantly less than achieved with the chemically defined reference medium for this cell line, product concentration was improved by 7% with the newly designed medium.

The most suitable formulation was selected for fine tuning in the third phase of medium development. Therefore, the concentrations of the component groups were varied and individual components as well as the buffer system were optimized. Vitamins, for example, had a profound effect on cell growth and cell specific productivity. The integral of viable cells was reduced with vitamin concentrations, while productivity increased. Vitamin concentrations above 35% were not suitable for cultivation as observed by diminished cell growth.

**Figure 1:**
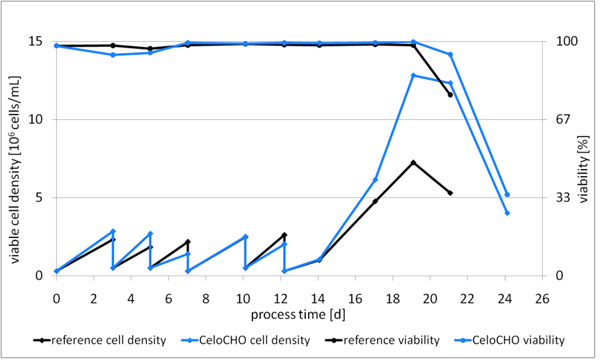
Adaptation of cell line producing IgG A to CeloCHO and batch experiment. The experiment was performed in shake flask scale without process optimization.

The final medium, CeloCHO, was tested with different CHO clones producing either IgG or fusion proteins. Figure 1 shows a batch experiment after adaptation of a recombinant cell line producing IgG A compared to the commercial available reference medium. Maximum viable cell density was improved 3-fold compared to the initial medium design with 12×10^6^ cells per ml and exceeded the performance of the chemically defined reference medium. The product concentration was improved by 14% in the batch experiment and 57% in the fed-batch experiment compared to the reference medium (Table 1). Product concentration was 1.4 g/L without further process optimization. The improvement in fed-batch indicated a good compatibility of basal and feed medium. The feed medium that was used for supplementation was also used for the development of the initial basal medium formulation, which was probably the reason for the good performance. Product concentration in fed-batch experiments with other CHO-K1 derived producer cell lines was improved between 6% and 25% demonstrating suitability for different CHO-K1 derived cell lines (Table 1).

**Table 1:** Relative product concentration in batch and fed-batch mode using CeloCHO with 4 different cell lines.

Cell line, CHO-K1 derived	IgG A, batch	IgG A, fed-batch	IgG B, fed-batch	IgG C, fed-batch
Reference medium^a^, Titer^b^	100%	100%	100%	100%
CeloCHO, Titer^b^	114%	157%	125%	106%

^a^ Chemically defined reference Medium optimized for each cell line. ^b^ Normalized to product concentration in reference medium.

The strategy for the development of a basal medium formulation based on a previously optimized feed solution proved suitable for medium development approaches without the need of extensive analytical work. Components were classified into five stock solutions based on chemical properties and combined as x-fold concentrates for fast media development. After a round of modifications regarding vitamins, buffer and salt optimization, cell densities up to 12×10^6^ cells per ml were achieved in batch experiments with the chemically defined, animal derived component free CHO medium. The product concentration in fed-batch experiments was improved up to 57% compared to the reference medium for the cell line producing IgG A. Data of the third phase of medium development delivered a starting point for medium optimization to clonal demands, since the effects of several key components were studied in detail. Cell growth and specific productivity, for example, could be modulated in a vitamin-dependent way. Six cell lines were studied for growth and recombinant protein production using CeloCHO and demonstrated feasibility for universal use.

